# Prenatal Influences on Size, Velocity and Tempo of Infant Growth: Findings from Three Contemporary Cohorts

**DOI:** 10.1371/journal.pone.0090291

**Published:** 2014-02-27

**Authors:** Costanza Pizzi, Tim J. Cole, Lorenzo Richiardi, Isabel dos-Santos-Silva, Camila Corvalan, Bianca De Stavola

**Affiliations:** 1 Cancer Epidemiology Unit, Department of Medical Sciences, University of Turin and CPO-Piemonte, Turin, Italy; 2 Centre for Statistical Methodology, London School of Hygiene and Tropical Medicine, London, United Kingdom; 3 Centre for Paediatric Epidemiology and Biostatistics, UCL Institute of Child Health, London, United Kingdom; 4 Non-Communicable Disease Epidemiology Department, London School of Hygiene and Tropical Medicine, London, United Kingdom; 5 Institute of Nutrition and Food Technology, University of Chile, Santiago de Chile, Chile; Johns Hopkins Bloomberg School of Public Health, United States of America

## Abstract

**Background:**

Studying prenatal influences of early life growth is relevant to life-course epidemiology as some of its features have been linked to the onset of later diseases.

**Methods:**

We studied the association between prenatal maternal characteristics (height, age, parity, education, pre-pregnancy body mass index (BMI), smoking, gestational diabetes and hypertension) and offspring weight trajectories in infancy using SuperImposition by Translation And Rotation (SITAR) models, which parameterize growth in terms of three biologically interpretable parameters: *size*, *velocity* and *tempo*. We used data from three contemporary cohorts based in Portugal (GXXI, n = 738), Italy (NINFEA, n = 2,925), and Chile (GOCS, n = 959).

**Results:**

Estimates were generally consistent across the cohorts for maternal height, age, parity and pre-pregnancy overweight/obesity. Some exposures only affected one growth parameter (e.g. maternal height (per cm): 0.4% increase in *size* (95% confidence interval (CI):0.3; 0.5)), others were either found to affect *size* and *velocity* (e.g. pre-pregnancy underweight vs normal weight: smaller *size* (−4.9%, 95% CI:−6.5; −3.3), greater *velocity* (5.9%, 95% CI:1.9;10.0)), or to additionally influence *tempo* (e.g. pre-pregnancy overweight/obesity vs normal weight: increased *size* (7.9%, 95% CI:4.9;10.8), delayed *tempo* (0.26 months, 95% CI:0.11;0.41), decreased *velocity* (−4.9%, 95% CI: −10.8;0.9)).

**Conclusions:**

By disentangling the growth parameters of *size*, *velocity* and *tempo*, we found that prenatal maternal characteristics, especially maternal smoking, pre-pregnancy overweight and underweight, parity and gestational hypertension, are associated with different aspects of infant weight growth. These results may offer insights into the mechanisms governing infant growth.

## Introduction

Birth size and early life growth trajectories have been found to be important predictors for the onset and development of a wide range of later diseases [1]–[6], with early postnatal weight gains becoming the focus of research into the development of overweight and obesity later in childhood and adulthood [7]–[11]. As a consequence there is also growing interest in prenatal predictors of rapid weight gain in infancy [12]–[13] and overweight and obesity later life [14]–[17].

A wide-ranging literature exists on the association of prenatal exposures - such as parental age, maternal, environmental and social factors, health status, life-style and pregnancy conditions, - with birth outcomes, mainly birth size and gestational age [18]–[22]. More recently, the association of these prenatal exposures with early life growth trajectories has also been investigated [13], [23]–[25], particularly with reference to features of postnatal rapid weight gain [12], [26], [27]. A limitation of most of these analyses is that they focus on relatively simple aspects of growth, such as differences in size at pre-specified age intervals. In addition such comparisons can only be performed when growth data are available at fixed time points and therefore may involve only a subset, possibly unrepresentative, of the original cohort [12].

In this paper we examine the association between several prenatal maternal exposures with weight trajectories of infants (0–2 years) from three recent cohorts based in countries with diverse socio-economic backgrounds (Portugal, Italy and Chile) using the shape-invariant random effects model called SuperImposition by Translation And Rotation (SITAR) [23], [28]. This approach allows the capture of individual trajectories, from irregularly spaced observations, through three parameters that have a direct biological interpretation - *size, velocity and tempo*. SITAR has been used before to model individual growth data [28]–[30], and is extended here to include multiple explanatory variables for each of its three parameters. The focus is on studying the prenatal influences on infant growth using data from different cohorts to evaluate the validity of the results, given the expected differences across the three source populations in distribution of the exposures as well as their correlations with potential confounders.

## Materials and Methods

All participants to the Generation XXI (GXXI), Nascita e INFanzia: gli Effetti dell'Ambiente (NINFEA) and Growth and Obesity Cohort Study (GOCS) cohorts have read and signed a written informed consent form. Data for this paper were analyzed anonymously. GXXI was approved by the Portuguese Data Protection Authority (CNPD - Comissão Nacional de Protecção de Dados). NINFEA study was approved by Ethical Committee of the San Giovanni Battista Hospital and CTO/CRF/Maria Adelaide Hospital of Turin (approval N. 0048362). GOCS study was approved by the Institutional Review Board of the Institute of Nutrition and Food Technology (INTA) of the University of Chile. Anonymized data are available upon request to qualified researchers for the purpose of academic, non-commercial, collaborative research.

### The cohort studies

#### GXXI

GXXI was established in 2005 in the Porto region of Portugal. All children born of women resident in the region and admitted to one of its five public hospitals for delivery, with a gestational age at birth greater than 24 weeks, were eligible to participate. Recruitment lasted from April 2005 to August 2006. Women were enrolled a few days before their due date and, the majority, completed baseline questionnaires between 24 and 72 hours after delivery. In total the baseline data consist of 8,311 singleton children. Children were actively followed-up through interviewer-administered questionnaires planned at 3, 6, 12–15 and 24 months of age. Due to logistic and financial constraints a restricted time window was allocated for each follow-up occasion and therefore it was not possible to interview every participant at each follow-up visit. The present analyses are based on the information collected at baseline and at the 2-years follow-up, which is available for 786 infants (9.5% of the original cohort). Maternal and birth characteristics of infants invited to participate in the 2-years follow-up were compared with the rest of the cohort. No systematic differences were found, suggesting that participants who were followed-up at 2 years of age are a representative sample of the whole cohort (Table S1); thus restriction of the analyses to this subgroup of subjects should not have biased the exposure-outcome estimates of interest. Further restrictions (subjects without follow-up weight measures (n = 3), subjects without gestational age data (n = 35) and subjects with missing data for the gender variable (n = 10)) were applied leading to 738 singleton babies included in the analyses. All growth data were retrieved prospectively from the child's health records by health professionals. These include anthropometric measures taken at birth and at about 1, 2, 4, 6, 9, 12, 15, 18 and 24 months of age, together with the actual dates of measurement. Up to 6 additional measurements and dates reported in the health records were also entered into the database. The median number of measurements per child is 10.

#### NINFEA

NINFEA is an on-going Italian web-based cohort study which started in 2005 and aims to recruit pregnant women via the Internet and follow up their children (more details in [31]–[32]). Enrollment is carried out at the study website (www.progettoninfea.it) where women complete the first questionnaire (Q1) at any time during their pregnancy. Active follow up is via online questionnaires administered at around 6 (Q2), 18 (Q3), 48 (Q4) months and 7 years (Q5) of age of the child. At Q2 women were asked to report the child's anthropometric measurements at birth, 3 and 6 months, while at Q3 they were asked to report the measures at 12 and 18 months. Revisions of these questionnaires, undertaken after approximately the first 1,500 mothers enrolled, led to inclusion of additional questions on the child's measures at the time of their completion. The analyses involve weight data from birth to around age 2, resulting in a median of 4 (range 1–7) measurements per child. These were obtained from the NINFEA database version 12.03 (downloaded in March 2012) and concern 2,925 singleton children with available data on gestational age at birth, whose mothers were born in Italy, and who, at the time of the data download, were eligible for at least the 6-months questionnaire (Q2). This implies that growth data up to about 6 months of age only are available for those infants who, at the time of the data download, were not eligible for the 18-months questionnaire (Q3). When comparing the distribution of baseline characteristics of infants with/without data from both Q2 and Q3 no systematic differences were observed (data not shown).

#### GOCS

GOCS is an on-going Chilean cohort aiming to study the association of early growth with children's maturation, adiposity and associated metabolic complications (more details in [33]). The study was initiated in 2006 when all children aged 2.6–4 years attending public nursery schools in six counties of Santiago were invited to participate if they were singleton births with a gestational age at birth between 37 and 42 weeks, and birth weight between 2500 and 4500 grams. Among the 1,498 eligible children 1,195 (80%) accepted the invitation. The analyses include all 959 children with exact gestational age data. Weight and height measurements from birth up to 36 months of age were extracted from routinely-completed health records; from the time of recruitment onwards, children were measured yearly at their nursery by a dietician. For these analyses only growth data up to around age 2 years were used, yielding a median of 6 (range 1–8) measurements per child. These include measures taken at birth and at about 1, 2, 4, 6, 12, 18 and 24 months of age. When comparing the distribution of baseline characteristics of infants with/without complete growth data, differences were observed with respect to maternal pre-pregnancy BMI, smoking and age at birth of the child (data not shown). Since these variables are included in the growth models described below, results can still be generalized to the full cohort under the assumption of missingness at random (see Methods section).

### Prenatal exposures

The following background maternal exposures were studied in relation to weight trajectories over the first 2 years of life: age, height, parity at the time of birth of the child and educational level. Pre-pregnancy body mass index (BMI), smoking status during pregnancy and pregnancy complications, namely gestational diabetes and pregnancy hypertension/eclampsia, were instead considered intermediate exposures as their values are likely to be affected by the background variables above. Data on prenatal variables were derived from questionnaires administered during pregnancy in NINFEA, at birth in GXXI, and when the children were approximately 3–4 years old in GOCS. Coding and further details are given in Table 1. Because of missing values a *core dataset* for each cohort was defined as the subset of records with complete information on the following core exposure variables: maternal height, age, education, parity, pre-pregnancy BMI and smoking status during pregnancy. The GXXI, NINFEA and GOCS *core datasets* include 605, 2,734 and 659 children respectively.

**Table 1:** Descriptive statistics of the main variables by cohort.

	GXXI (N = 738)		NINFEA (N = 2,925)		GOCS (N = 959)	
	*N* a	*%* b	*N* a	*%* b	*N* a	*%* b
**Child characteristics**						
Mean gestational age *(weeks ± SD)*	738	39.1±1.6	2,925	39.6±1.6	959	39.6±1.3
Gender						
*Female*	365	49.5	1,441	49.3	487	49.2
*Male*	373	50.5	1,484	50.7	472	50.8
**Maternal characteristics**						
Mean height *(cm* ± *SD)*	629	161.5±5.9	2,836	164.7±6.1	903	156.9±5.8
Mean age *(years* ± *SD)*	737	30.3±5.1	2,925	33.5±4.1	888	27.0±6.9
Parity c						
*Nulliparous*	462	62.9	2,105	74.1	373	58.1
*Parous*	272	37.1	737	25.9	517	41.9
*Missing*	*4*		*83*		*69*	
Educational level d						
*Low*	362	49.7	147	5.1	323	36.3
*Medium*	172	23.6	1,053	36.4	383	43.0
*High*	194	26.7	1,690	58.5	184	20.7
*Missing*	*10*		*35*		*69*	
Pre-pregnancy BMI						
*<18.5*	30	4.9	235	8.3	34	5.1
*18.5*–*24.99*	376	60.7	2,060	72.8	395	59.8
*25+*	213	34.4	533	18.9	232	35.1
*Missing*	*119*		*97*		*298*	
Smoke during pregnancy e						
*No*	574	79.5	2,632	91.6	809	91.0
*Up to 1st trimester*	53	7.3	51	1.8	80	9.0
*After 1st trimester*	95	13.2	190	6.6	–	–
*Missing*	*16*		*52*		*70*	
Pregnancy complications f						
Gestational diabetes						
*No*	560	92.3	2,506	92.0	913	95.2
*Yes*	47	7.7	218	8.0	46	4.8
*Missing*	*131*		*201*		*0*	
Hypertension/eclampsia						
*No*	576	95.2	2,498	91.8	878	91.6
*Yes*	29	4.8	222	8.2	81	8.4
*Missing*	*133*		*205*		*0*	

a Total N might vary across variables due to missing values

b Percentages are computed based on the total number of non-missing values

c In GOCS child order is used as a proxy for parity

d GXXI: Low = ≤9 years, Medium = 9–12 years, High = Degree or more; NINFEA: Low = None/Primary/Secondary school, Medium = High school, High = Degree or more; GOCS: Low = None/Primary/Secondary school, Medium = High school, High = High School + technical education or more

e In GOCS smoking during pregnancy is categorized as No/Rarely vs Frequently

f Mothers suffering from these diseases before pregnancy (information available only in GXXI and NINFEA) classified as “No”

### Statistical methods

#### SITAR model

The observed weight trajectories were modelled using a recently developed shape invariant random effects model. It was introduced by Cole [28] to study height trajectories in puberty, following the model proposed by Beath to analyse weight growth in infancy [23]. Let *y_it_* be the weight of child *i* at age *t*, then SITAR is specified as:
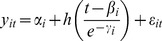
(1)where *h(z)* is a natural cubic spline of transformed age *z*, *α_i_*, *β*_i_ and *γ_i_* are subject-specific growth parameters, and ε_it_ is the residual error term assumed to have mean zero and constant variance. The three parameters correspond respectively to the *size*, *tempo* and *velocity* of growth specific to each child: *α_i_* (*size*) represents the shift in the weight axis, while *β*_i_ (*tempo*) and *γ_i_* (*velocity*) represent the change in location and scale to be applied to the age axis, respectively, in order for all children to share the same shape (mean spline curve *h(z)*). *Size* (*α_i_*) is expressed in units of weight, *tempo* (*β*_i_) in units of age, while *velocity* (*γ_i_*) is a multiplier, and therefore is scale-free and reported as a percentage. Pizzi et al [34] discuss in detail how these parameters are to be interpreted given their close correlations. In brief they can be parameterized as follows: let *α_i_ = α_0_+α_1i_*, where *α_0_* is a fixed parameter, representing the size of a reference child, and *α_1i_* a random, normally distributed variable with mean zero and constant variance, and let similar specifications for *β*_i_ and *γ_i_*, then estimation can be carried out by maximum likelihood as for any (non-linear) mixed effects model [35]. Irregular observations can be handled under the assumption of missing at random (MAR) [36]. From a biological perspective *α_1i_* (*size*) will be positive for heavier children, while *β*_1i_ (tempo) is related to the timing of maximum growth velocity and therefore will be negative for children whose growth is more advanced at earlier ages (earlier velocity peak), and *γ_1i_* (*velocity*) will be positive for children with faster growth [28].

A covariate X with observed value x_i_ on subject *i* can be included in the model by specifying the three growth parameters as follow:
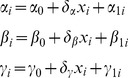
(2)where *δ_α_*, *δ_β_* and *δ_γ_* represent the contribution of the covariate to a child's *size*, *tempo* and *velocity*, respectively. Generalization of equation (2) to multiple covariates is straightforward. This is a slightly different parameterization from the one adopted by Beath [23].

#### Analyses

Weight was log-transformed to aid meeting the distributional assumptions of the model. As a consequence δ_α_ is to be interpreted as percentage changes in size relative to the reference child [37]. Age was measured in months, hence δ_β_ is also expressed in months. The spline function *h(z)* was defined by placing the internal knots at quantiles of the age distribution, appropriate for each cohort because of varying richness and spread of the available weight measurements (four knots were used for analyses of GXXI and GOCS data and three for analyses of NINFEA). The complexity of the SITAR model relatively to the available data led to imposing constraints on its parameters, namely that the *tempo* of the standard child, *β_0_*, was zero. Furthermore to be able to compare the three cohorts, *δ_β_*, the contribution of each covariate on a child's *tempo*, was also constrained to be zero. These constraints were relaxed in analyses specific to GXXI as it had more weight growth measurements.

Models were initially fitted separately by study. We first included one explanatory variable at a time, with adjustment by gender and gestational age (we will refer to the latter results as “minimally-adjusted estimates”). We used all available data and also just the *core datasets* to allow comparisons between unadjusted and adjusted estimates for each of these variables. Fully-adjusted estimates were obtained by fitting two separate models to the *core datasets*: (i) the background explanatory variables were mutually adjusted, as well as adjusted for gestational age and gender; (ii) the intermediate explanatory variables were mutually adjusted, as well as adjusted for the background variables, gestational age and gender.

Models were also refitted on the pooled data from the three cohorts, including a fixed effect for the study indicators and assessing evidence of heterogeneity via significance tests of the interaction between each covariate and the study indicators (one covariate at a time, using the Wald test).

## Results

### Descriptive results

There is considerable variation in the distribution of the prenatal exposures across the three birth cohorts (Table 1); in particular, Chilean and Portuguese mothers are on average 8 and 3 cm shorter, and 6 and 3 years younger at birth, respectively, than their Italian counterparts. Despite being on average younger, the proportion of multiparous mothers is higher among GOCS participants. Educational level strongly differs across cohorts, with only 5% of the NINFEA mothers being in the lowest educational category as opposed to 36% in GOCS and almost 50% in GXXI, and with almost 60% highly educated women in NINFEA compared to 27% and 20% in GXXI and GOCS, respectively. Because of the study design, education is a strong predictor of participation into NINFEA [32], and this explains many of the differences observed. The prevalence of overweight/obese women is much lower in NINFEA, while prevalence of underweight is slightly higher. Approximately 20% of GXXI women smoked during pregnancy with the corresponding figure in the other two populations below 10%. Gestational diabetes was less frequently diagnosed in GOCS, while gestational hypertension/eclampsia was less frequently diagnosed in GXXI. These differences in baseline characteristics are consistent with the differences in demographic and socio-economic distribution of the three source populations as well as in the study design of the three cohorts. This heterogeneity allowed us to evaluate the validity of the findings, as homogeneous covariate effects estimated from populations with different confounding structures would indicate minimal residual confounding.

### Explanatory variables for size and velocity

#### Cohort-specific analyses

Table 2 presents the estimated minimally-adjusted and fully-adjusted covariate-specific parameters (i.e. the relevant *δ_α_* and *δ_γ_*), by cohort, obtained from models fitted to the *core datasets*. The minimally-adjusted estimates obtained when fitting the models to each whole cohort are reported in Table S2: they are generally close to the minimally-adjusted estimates in Table 2 indicating that the *core datasets* are likely to be representative of the corresponding whole cohorts. The minimally-adjusted and fully-adjusted estimates in Table 2 are very similar, indicating little reciprocal confounding among these variables. Despite some between-cohort differences, the findings overall are consistent with *size* (*α_i_*) being positively associated with maternal height (NINFEA, fully-adjusted: δ_α_ = 0.4%; similarly in the other cohorts), pre-pregnancy overweight/obesity (NINFEA, fully-adjusted: δ_α_ = 2.1%; similarly in the other cohorts) and parity (GXXI, fully-adjusted: δ_α_ = 4.5%; similarly in NINFEA), but negatively associated with smoking during pregnancy (fully-adjusted: δ_α_≈−3% in GXXI and NINFEA) and maternal pre-pregnancy underweight (fully-adjusted: δ_α_≈−4% in each cohort). Post-natal growth *velocity* (*γ_i_*) was positively associated with maternal smoking (GXXI, fully-adjusted: δ_γ_ = 13.2%; NINFEA, fully-adjusted: δ_γ_ = 6.5%), and possibly maternal underweight (NINFEA, fully-adjusted: δ_γ_ = 4.4%), but negatively associated with parity (GXXI, fully-adjusted: δ_γ_ = −6.1%; similarly in NINFEA). The results for education were heterogeneous: while in GXXI medium/highly educated women have bigger children who tend to have slower growth velocity, and in GOCS the children from less educated mothers have slower growth compared to those in the reference group, in NINFEA no association was found.

**Table 2:** Estimated coefficients and 95% confidence interval for the association between covariates and size and velocity parameters by cohort.

	GXXI (N = 605)								NINFEA (N = 2,734)								GOCS (N = 659)							
	Minimally-adjusted a				Fully-adjusted b				Minimally-adjusted a				Fully-adjusted b				Minimally-adjusted a				Fully-adjusted b			
	Size		Velocity		Size		Velocity		Size		Velocity		Size		Velocity		Size		Velocity		Size		Velocity	
	*%*	*95%CI*	*%*	*95%CI*	*%*	*95%CI*	*%*	*95%CI*	*%*	*95%CI*	*%*	*95%CI*	*%*	*95%CI*	*%*	*95%CI*	*%*	*95%CI*	*%*	*95%CI*	*%*	*95%CI*	*%*	*95%CI*
**Background**																								
Maternal height	0.4	0.2; 0.5	0.2	−0.2; 0.6	0.3	0.2; 0.5	0.2	−0.2; 0.6	0.4	0.3; 0.5	0.02	−0.2; 0.2	0.4	0.3; 0.5	0.1	−0.1; 0.2	0.4	0.2; 0.5	−0.03	−0.6; 0.1	0.4	0.2; 0.5	−0.3	−0.7;0.02
Maternal age	0.1	−0.1; 0.3	0.2	−0.3; 0.7	−0.1	−0.3;0.1	0.5	0.0; 1.0	0.1	−0.0;0.2	−0.2	−0.5; 0.1	0.02	−0.1; 0.1	−0.1	−0.3; 0.2	0.1	0.01;0.2	−0.2	−0.4; −0.1	0.1	0.0; 0.3	−0.1	−0.4;0.3
Maternal parity c																								
*Nulliparous*	0	–	0	–	0	–	0	–	0	–	0	–	0	–	0	–	0	–	0	–	0	–	0	–
*Parous*	3.4	1.2; 5.5	−3.5	−8.4; 1.5	4.5	2.2;6.8	−6.1	−11.6; −0.6	3.0	2.0; 4.0	−5.6	−8.2; −3.1	2.8	1.8; 3.9	−5.8	−8.4; −3.2	2.0	0.3; 3.6	−3.7	−7.4; 0.3	1.0	−1.0;2.9	−2.1	−6.7;2.6
Maternal education^d^																								
Low	0	–	0	–	0	–	0	–	−1.5	−3.7; 0.8	2.9	−2.7; 8.6	−0.4	−2.5; 1.7	2.8	−2.6; 8.1	0.7	−1.1; 2.5	−5.7	−10.0; −1.4	0.6	−1.2;2.4	−5.5	−9.9; −1.1
*Medium*	3.3	0.7; 5.9	−0.4	−6.4; 5.6	3.3	0.8; 5.9	−1,2	−7.3; 4.8	0	–	0	–	0	–	0	–	0	–	0	–	0	–	0	–
*High*	2.3	−0.1; 4.8	−2.4	−8.1; 3.2	2.9	0.4; 5.5	−5.4	−11.4; 0.6	−0.6	−1.6; 0.4	−0.6	−3.0; 1.9	−0.8	−1.7; 0.2	−0.7	−3.0; 1.6	−0.7	−2.8; 1.5	−0.9	−5.9; 4.2	−1.2	−3.3;0.9	−0.5	−5.6;4.6
**Intermediate**																								
Pre-pregnancy BMI																								
*<18.5*	−4.4	−9.2; 0.4	2.5	−8.7;13.6	−4.2	−8.9;0.5	−0.7	−11.8;10.4	−4.4	−6.0; −2.8	5.2	1.2; 9.2	−4.1	−5.7; −2.6	4.4	0.4; 8.3	−3.6	−7.2; 0.1	−5.9	−14.6; 2.9	−4.3	−7.8; −0.1	−4.5	-13.4;4.3
*18.5–24.99*	0	–	0	–	0	–	0	–	0	–	0	–	0	–	0	–	0	–	0	–	0	–	0	–
*25+*	3.4	1.2; 5.6	0.9	−4.1; 5.9	4.0	1.8; 6.1	0.7	−4.3; 5.8	2.0	0.9; 3.1	−1.6	−4.4; 1.2	2.1	0.9; 3.2	−2.1	−4.9; 0.7	1.7	−0.0;3.4	−1.4	−5.4; 2.6	1.8	0.04; 3.5	−0.3	−4.5;3.9
Maternal smoking^e^																								
*No*	0	–	0	–	0	–	0	–	0	–	0	–	0	–	0	–	0	–	0	–	0	–	0	–
≤*1^st^ trimester*	−2.8	−6.9;1.3	6.6	−2.7;15.9	−1.4	−5.3;2.5	6.6	−2.7; 15.9	0.7	−2.9; 4.2	4.1	−4.7;12.8	0.2	−3.1; 3.5	4.6	−3.7;12.9	1.2	−1.7; 4.1	−7.4	−14.1; −0.7	0.6	−2.2; 3.5	−5.1	−12.0;1.8
*>1^st^ trimester*	−3.8	−6.8; −0.7	12.8	5.8;19.8	−2.9	−5.8;0.1	13.2	6.1; 20.3	−3.2	−5.1; −1.3	8.4	3.6;13.2	−2.6	−4.3; −0.9	6.5	2.0; 10.9								

a Estimates derived from model adjusted for gender and gestational age fitted on the sample of data with no missing values for the following maternal variables: height, age, parity, educational level, pre-pregnancy BMI and smoking during pregnancy

b Background variables are mutually adjusted and further adjusted for gender and gestational age; intermediate variables are mutually adjusted and further adjusted for background variables, gender and gestational age

c In GOCS child order was used as a proxy for parity

d GXXI: Low = ≤9 years, Medium = ≤12 years, High = Degree or higher; NINFEA: Low =  ≤Secondary school, Medium = High school, High = Degree or higher; GOCS: Low =  None/Primary/Secondary school, Medium = High school, High = High School + technical education or higher

e In GOCS smoking during pregnancy was categorized as No/Rarely vs Frequently

The model that examined pregnancy complications showed that, when fully adjusted for the other characteristics, gestational diabetes was not associated with infant weight growth (Table 3). In contrast, children from mothers with gestational hypertension were smaller and with a steeper growth curve (GXXI, fully-adjusted: δ_α_ = −6.4%, δ_γ_ = 12.8%; similarly in NINFEA), although this pattern was not present in GOCS.

**Table 3:** Estimated coefficients and 95% confidence interval for the association between pregnancy complications and size and velocity parameters by cohort.

	GXXI (N = 492)								NINFEA (N = 2,523)								GOCS (N = 659)							
	Minimally-adjusted a				Fully-adjusted b				Minimally-adjusted a				Fully-adjusted b				Minimally-adjusted a				Fully-adjusted b			
	Size		Velocity		Size		Velocity		Size		Velocity		Size		Velocity		Size		Velocity		Size		Velocity	
	*%*	*95%CI*	*%*	*95%CI*	*%*	*95%CI*	*%*	*95%CI*	*%*	*95%CI*	*%*	*95%CI*	*%*	*95%CI*	*%*	*95%CI*	*%*	*95%CI*	*%*	*95%CI*	*%*	*95%CI*	*%*	*95%CI*
Diabetes	0.8	−3.8;5.3	4.6	−5.9;15.0	0.6	−3.8;4.9	5.7	−4.5;16.0	2.0	0.3;3.7	−5.1	−9.3; −0.9	1.5	−0.3;3.3	−3.1	−7.6;1.3	0.6	−3.1;4.3	4.1	−4.7;12.8	−0.6	−4.2;3.1	7.0	−1.8; 15.8
Hypertension	−4.9	−10.6;5.5	12.3	−0.6;25.2	−6.4	−11.7; −1.0	12.8	0.3; 25.3	−3.8	−5.5; −2.0	8.7	4.4;13.0	−4.5	−6.2; −2.7	9.0	4.7;13.4	2.2	−0.7;5.1	−3.8	−10.6;3.0	1.3	−1.4;4.2	−4.0	−10.9; 2,9

a Estimates derived from model adjusted for gender and gestational age fitted on the sample of data with no missing values for the following maternal variables: height, age, parity, educational level,pre-pregnancy BMI, smoking during pregnancy, gestational diabetes and gestational hypertension

b Adjusted by sex, gestational age and maternal height, age, parity, educational level, pre-pregnancy BMI and smoking during pregnancy

#### Pooled analyses

Pooled analyses of the three cohorts show significant heterogeneity of effects for some covariates (smoking, gestational hypertension and gestational diabetes), with the differences arising from GOCS, unsurprisingly given the results of Tables 2–3, the retrospective collection of its prenatal data and its inclusion criteria. As there was no evidence of heterogeneity between GXXI and NINFEA, their data were pooled with results reported in Table 4 (only the fully adjusted estimates are reported). The estimated coefficients for pre-pregnancy BMI confirm that babies from underweight mothers are smaller but with a greater postnatal growth rate (i.e. *velocity* (*γ_i_*)), while children from overweight/obese women have a bigger *size* (*α_i_*) without evidence of decreased postnatal growth rate. Results for maternal education show that less educated mothers have smaller children that however have the same growth *velocity* (*γ_i_*) as children of more educated mothers (Table 4).

**Table 4:** Fully-adjusted estimated coefficients and 95% confidence interval for the association between covariates and size and velocity parameters on the pooled GXXI & NINFEA datasets.

	GXXI+NINFEA (N = 3,339) a			
	Size		Velocity	
	*%*	*95%CI*	*%*	*95%CI*
**Background** b				
Maternal height	0.4	0.3; 0.5	0.1	−0.1; 0.2
Maternal age	0.02	−0.1; 0.1	0.02	−0.2; 0.3
Maternal parity				
*Nulliparous*	0	–	0	–
*Parous*	3.1	2.1; 4.1	−5.5	−8.0; −3.0
Maternal education c				
Low	−2.2	−3.7; −0.6	2.4	−1.4; 6.2
*Medium*	0	–	0	–
*High*	−0.9	−1.8; 0.1	−0.7	−3.0; 1.6
**Intermediate** d				
Pre-pregnancy BMI				
*<18.5*	−4.9	−6.5; −3.3	5.9	1.9; 10.0
*18.5–24.99*	0	–	0	–
*25+*	2.4	1.4; 3.5	−0.5	−3.1; 2.1
Maternal smoking				
*No*	0	–	0	–
≤*1^st^ trimester*	−0.9	−3.4; 1.7	5.9	−0.5; 12.3
*>1^st^ trimester*	−3.3	−4.8; −1.7	10.2	6.3; 14.3
	(N = 3,015) e			
Gestational diabetes	1.1	−0.6; 2.7	−0.8	−5.0; 3.3
Gestational hypertension	−5.2	−6.9; −3.5	10.6	6.4; 14.8

a Model fitted on the sample of data with no missing values for the following maternal variables: height, age, parity, educational level, pre-pregnancy BMI and smoking during pregnancy

b Background variables are mutually adjusted and further adjusted for gender and gestational age

c GXXI: Low = ≤9 years, Medium = ≤12 years, High = Degree or higher; NINFEA: Low =  ≤Secondary school, Medium = High school, High = Degree or higher

d Intermediate variables are mutually adjusted and further adjusted for background variables, gender and gestational age

e Model fitted on the sample of data with no missing values for the maternal variables: height, age, parity, educational level, pre-pregnancy BMI, smoking during pregnancy, gestational diabetes and gestational hypertension

In order to examine whether the observed heterogeneity across the cohorts was due to differences in entry criteria, we replicated cohort-specific analyses on the subset of GXXI and NINFEA children who were born at term and with a birth weight of 2500–4500 grams, using the same entry criteria as GOCS. The results pointed to much more similar effects across the cohorts.

### Explanatory variables for size, velocity and tempo

Finally we rerun the analyses allowing for covariate effects on *tempo*, restricting them to the GXXI cohort because of its rich number of repeated weight observations (similar analyses for the other cohorts failed to converge). The results are reported in Table 5 (only the fully adjusted estimates are reported). There is no evidence of an effect of maternal height, age, pre-pregnancy underweight or smoking on *tempo* (*β*_i_), and therefore no change in the estimated effects on *size* (*α_i_*) or *velocity* (*γ_i_*). However parity, pre-pregnancy overweight/obesity, and hypertension do influence *tempo* (*β*_i_) of growth. Infants of parous mothers have relatively earlier growth spurts by about 5 days (δ_β_ = −0.17 months, 95% CI -0.34; −0.01). Allowing for this association ‘explains away’ some of the earlier associations found between parity and *size* (*α_i_*) and *velocity* (*γ_i_*) (both are substantially reduced; see Table 2 and Table 5). In contrast infants have delayed *tempo* (*β*_i_) by about 8 days (δ_β_ = 0.26, 95% CI 0.11; 0.41) if their mother is overweight/obese. As for parity, given the correlations among the three growth parameters, including maternal overweight/obesity in the specification of *tempo* (*β*_i_) changes its association with *size* (*α_i_*) and *velocity* (*γ_i_*). In particular that for *velocity* becomes negative (δ_γ_ = −4.9, 95% CI −10.8; 0.9) implying that infants of overweight/obese mothers not only have a later peak, but also have slower velocity than that of a reference child. For hypertension the association with *size* (*α_i_*) and *velocity* (*γ_i_*) is reduced when an association with *tempo* (*β*_i_) is allowed. The latter is found to be positive (δ_β_ = 0.31, 95% CI 0.08; 0.53) indicating a delay in peak velocity of almost 10 days (Table 5).

**Table 5:** Fully-adjusted estimated coefficients and 95% confidence interval for the association between covariates and size, tempo and velocity parameters on the GXXI data.

	GXXI (N = 605) a							
	Size			Tempo			Velocity	
	*%*		*95%CI*	*β* b		*95%CI*	*%*	*95%CI*
**Background** c								
Maternal height	0.5		0.2; 0.7	0.01		−0.01; 0.02	−0.05	−0.5; 0.4
Maternal age	−0.1		−0.4; 0.2	−0.01		−0.02; 0.01	0.6	0.01; 1.2
Maternal parity								
*Nulliparous*	0		–	0		–	0	–
*Parous*	1.0		−2.2; 4.3	−0.17		−0.34; −0.01	−1.1	−7.4; 5.2
Maternal education d								
Low	0		–	0		–	0	–
*Medium*	3.6		0.1; 7.2	0.003		−0.18; 0.19	−2.2	−9.2; 4.7
*High*	3.6		0.1; 7.1	0.08		−0.11; 0.26	−7.0	−13.9; −0.1
**Intermediate** e								
Pre-pregnancy BMI								
*<18.5*	−5.9		−12.3; 0.5	−0.09		−0.41; 0.23	2.6	−10.2; 15.4
*18.5–24.99*	0		–	0		–	0	–
*25+*	7.9		4.9; 10.8	0.26		0.11; 0.41	−4.9	−10.8; 0.9
Maternal smoking								
*No*	0		–	0		–	0	–
≤*1^st^ trimester*	−0.9		−6.4; 4.5	−0.02		−0.29; 0.25	6.2	−4.6; 16.9
*>1^st^ trimester*	−2.8		−6.9; 1.3	−0.08		−0.28; 0.12	14.0	5.8; 22.2
	(N = 492) f							
Gestational diabetes	3.1	−3.9; 10.1			0.06	−0.13; 0.24	0.7	−12.7; 14.2
Gestational hypertension	−3.5	−12.2; 5.3			0.31	0.08; 0.53	8.7	−7.9; 25.3

a Model fitted on the sample of data with no missing values for the following maternal variables: height, age, parity, educational level, pre-pregnancy BMI and smoking during pregnancy

b Model is on the log-weight and age scales, thus the effect on tempo is on the age unit (months)

c Background variables are mutually adjusted and further adjusted for gender and gestational age

d Low = ≤9 years, Medium = ≤12 years, High = Degree or higher

e Intermediate variables are mutually adjusted and further adjusted for the background variables, gender and gestational age

f Model fitted on the sample of data with no missing values for the following maternal variables: height, age, parity, educational level, and pre-pregnancy BMI; smoking during pregnancy, gestational diabetes and gestational hypertension

**Figure 1:**
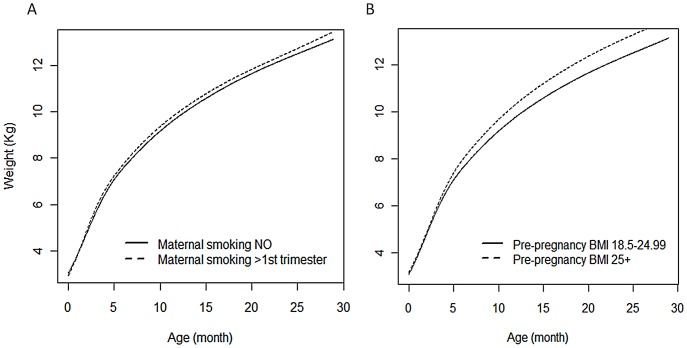
Predicted weight curves corresponding to the effect sizes reported in Table 5. A. Predicted weight curves for different categories of maternal smoking during pregnancy. The predicted curve for children exposed to maternal smoking during pregnancy beyond the 1^st^ trimester lies below that of those not exposed for the first months of life, but then lies above after 4–5 months of life due to their increased velocity. B. Predicted weight curves for different categories of maternal pre-pregnancy BMI. The predicted curve for children of overweight/obese mothers lies above that of children of mothers with a pre-pregnancy BMI between 18.5 and 25, with the difference between the two curves increasing with time.

To illustrate the effect sizes reported in Table 5, graphs of the trajectories predicted for children with different combinations of covariates are displayed in Figure 1. The left graph shows the predicted weight of infants whose mother did/did not smoke during pregnancy after the first trimester, holding all the other predictors constant (Figure 1 A). The graph on the right shows the predictions for infants whose mother was normal weight compared to overweight/obese before the index pregnancy (Figure 1 B). In both settings the curve in exposed children is higher (especially after 5 months of life) than in the non exposed, with predicted weight at 24 months, for example, of 12.3 kg when non exposed and 12.5 kg when exposed to maternal smoking. Similarly the predicted weight at 24 months is 12.3 kg when not exposed to maternal overweight/obesity and 13.1 kg when exposed. Hence, although some of the effect sizes estimated for each growth parameter are generally relatively small, when taken together they reveal interesting differences in predicted trajectories, with the actual effect estimates adding insights into the specific aspects of growth (i.e. *size*, *velocity* and *tempo*) that are influenced by these prenatal factors.

## Discussion

In this paper we investigated prenatal influences on weight growth in infancy in order to contribute to the understanding of its role in the development of a wide range of later diseases. We used data on children belonging to three contemporary cohorts based in Portugal, Italy and Chile in order to compare effects across socio-economically and geographically diverse populations and gain a more robust understanding of these associations, while accounting for potentially different confounding patterns. The individual weight trajectories were modelled using SITAR [28], a model that provides biologically interpretable growth parameters, extended here to include multiple explanatory variables.

Our analyses indicate that prenatal exposures affect different dimensions of the weight trajectories. In all cohorts, *size* was positively associated with maternal height, parity and pre-pregnancy overweight/obesity, and negatively with pre-pregnancy underweight. Additionally in all cohorts parity negatively affected *velocity*. In contrast, only for infants from the two European studies, maternal smoking and gestational hypertension were associated with reduced *size* and increased *velocity*, while pre-pregnancy underweight was positively associated with *velocity*. Maternal education was only a moderate predictor of *size* in the European cohorts and of *velocity* in the Chilean cohort. When *tempo* was modeled in terms of covariates in analyses restricted to GXXI, we found that part of the impact on *size* and *velocity* observed for parity, maternal overweight/obesity and hypertension was captured by their influence on the *tempo* dimension. In particular, infants of parous mothers were found to have an earlier timing of growth, while those of overweight/obese or gestational hypertensive mothers to have it delayed. We found instead no evidence of an effect of maternal height, age, or smoking on *tempo*.

While some of these results are not new - e.g. the relation between parity [13], [24], [26] and smoking [13]–[14] with infant *size* and weight *velocity*, the positive association between maternal overweight/obesity with increased *size* (which corroborates the existing evidence on an intergenerational transmission of obesity [17]) - other findings are of interest, in particular the association between gestational hypertension and reduced *size*, delayed *tempo* and increased *velocity*, and the effect of maternal underweight on *size* and *velocity*. The former is consistent with current evidence of an association of hypertension with fetal growth retardation [38]. For the latter, while the consequences of maternal obesity have been extensively investigated, less evidence is currently available on the effect of maternal pre-pregnancy underweight, especially on postnatal growth rate in economically developed countries. What we found in the European cohorts is that maternal underweight was associated with reduced *size* and increased *velocity*, while in the Chilean cohort only an effect on *size* was observed. We also found only a weak association between gestational diabetes and *size* in GXXI and NINFEA, despite previous findings linking it with increased birth weight and adiposity later in life [17], [39]. This is possibly due to the self-reported and coarse (i.e. no distinction in severity) nature of the information available in all three cohorts.

A strength of these combined results is that they are derived from modelling the joint association of multiple exposures on multiple growth parameters simultaneously. Another strength of the approach adopted in this paper is that we used all the available growth data (assuming that the frequency and timing of the observations do not depend on the values that are not observed, i.e. that data are MAR [36]). This is in contrast to the most common approach used in the epidemiological literature to analyse growth data which consists of comparing anthropometric measures taken at two fixed time points across subgroups of children (e.g. those defined by maternal characteristics). Such comparisons can only be performed for participants with observations at both occasions, therefore involving only a subset of the original cohort which leads to unbiased results only if missingness is completely at random [12]. Specifications of mixed effects models other than SITAR have been used to study growth data that are irregularly spaced, such as linear splines models [40]. Similarly to SITAR they require MAR [36]. However, they are not as flexible in modelling non-linear growth (linear mixed models) or not as interpretable (linear splines models) as SITAR. More specifically, the advantage of SITAR is the ability to naturally deal with the non-linear shape of the weight trajectories - via the use of a cubic spline - and to summarize the growth process via three biologically meaningful parameters, two of which - *velocity* and *tempo* - separate the growth rate into specific components when trajectories are non-linear. This has given us insights into what governs the timing of peak growth velocity in infancy when we were able to fit the expanded model with explanatory variables for *tempo*, as well as *size* and *velocity*. Moreover our study showed that SITAR can be successfully fitted to dataset with relatively sparse data, such as NINFEA, providing results consistent with those obtained with richer datasets. However, when examining the association between prenatal factors and growth, we had to impose some constraints allowing for an effect on *size* and *velocity* only, as the model also including a tempo effect failed when fitted to the NINFEA and GOCS data. This is likely to be due to lack of heterogeneity in GOCS, which only include term children, and to lack of sufficient growth observations in NINFEA. The fully specified model was instead successfully fitted to the GXXI cohort, which has the greater number of weight growth measurements.

As stated above, the motivation for the inclusion of data from three different cohorts was the evaluation of the validity of results across different settings, as also advocated by other researchers in the field (see a recent paper published within the framework of the CHICOS project [41]). Because the cohorts are based in countries with diverse socio-economic backgrounds (Portugal, Italy and Chile), they are likely to be affected by different confounding. Furthermore their data arise from different study designs, in particular with NINFEA being an internet based cohort with growth data reported by the mothers and GOCS having all the exposure data used here collected retrospectively. Despite this, the results for maternal height, maternal overweight/obesity and parity show homogeneous effects across the cohorts, indicating that residual confounding for the effect of these variables is unlikely. There were however some heterogeneous results across the three cohorts in relation to the effect of maternal smoking and hypertension, with the results for GOCS differing from those of the other two cohorts. Some of the differences were due to the GOCS inclusion criteria, with possibly also a contribution from differences in quality and coarseness of the available data, in particular in relation to pregnancy complications. Fortunately, these differences did not lead to cross-cohort heterogeneous results for maternal pre-pregnancy BMI, despite having been recorded when the children were approximately 3–4 years old. It is also reassuring that these results are in line with previous findings, as stated above.

We have not examined the effect of early postnatal factors such as breastfeeding because such investigations would have involved mediation analysis and this was beyond the scope of this paper. Understanding the pathways from prenatal to postnatal – mediatory – factors to growth trajectories require careful formulation of causal questions and the adoption of appropriate analytical methods [42]. This is the focus of current work.

In summary, our findings are that growth trajectories in contemporary infants from economically and geographically diverse countries such as Portugal, Italy and Chile share some common features, in particular with respect to the effect of maternal height, maternal overweight/obesity and parity. In the two European cohorts we also found interestingly separate effects of maternal underweight, smoking and hypertension on the child's *size* and *velocity*, and when growth data were rich and the effect on *tempo* could also be examined, we found that parity, maternal overweight/obesity and gestational hypertension had interesting effects on the timing of growth. Our analytical approach therefore succeeded in separating the relationships between prenatal maternal characteristics and infant growth into different components, and may inform new biological insights into the mechanisms governing infant growth.

## Supporting Information

Table S1 **GXXI: Baseline characteristics stratified by 2-year follow-up participation.** Comparison of maternal and birth characteristics of infants invited to participate in the 2-years follow-up vs the rest of the cohort.
(PDF)

Table S2 **“Minimally-adjusted” estimated coefficients and 95% confidence interval for the association between covariates and size and velocity parameters by cohorts.** Gender- and gestational age-adjusted estimates of the association between covariates and size and velocity parameters obtained fitting the models to each whole cohort.
(PDF)
