# Supplementary material for: Prenatal Influences on Size, Velocity and Tempo of Infant Growth: Findings from Three Contemporary Cohorts
Source: PLoS One. 2014 Feb 27;9(2):e90291. doi: 10.1371/journal.pone.0090291 (PMC3937389; doi:10.1371/journal.pone.0090291)
Supplement: Table S1 — GXXI: Baseline characteristics stratified by 2-year follow-up participation. Comparison of maternal and birth characteristics of infants invited to participate in the 2-years follow-up vs the rest of the cohort. (PDF) [file pone.0090291.s001.pdf]

TABLE S1. GXXI: Baseline characteristics stratified by 2-year follow-up participation

	**With 2-year follow-up (n=786)**		**Without 2-year follow-up (n=7,525)**	
	Mean ± SD or %	*N*	*Mean ± SD or %*	*N*
Maternal characteristics				
Age (yr)	30.4 ± 5.0	785	29.4 ± 5.7	7,512
Height (cm)	161.6 ± 5.9	670	160.6 ± 6.2	5,767
Pre-pregnancy weight (kg)	62.2 ± 11.7	750	61.6 ± 11.4	7,321
Pregnancy weight gain (kg)	13.0 ± 5.1	585	12.9 ± 5.5	5,853
High educational level [a]	26.7 %	207	22.1%	1,621
Have a partner	94.4%	736	93.4%	6,996
Employed	76.5%	530	70.0%	5,093
Partner employed	94.1%	622	93.8%	6,535
Nulliparous	62.5%	489	59.4%	4,444
Use of infertility treatment	2.5%	19	1.7%	129
Smoking during pregnancy	20.1%	154	23.7%	1,761
Child's characteristics				
Birth weight (kg)	3.19 ± 0.48	758	3.19 ± 0.49	7,394
Birth length (cm)	48.9 ± 2.3	724	48.8 ± 2.2	7,062
Birth head circumference (cm)	34.3 ± 1.5	697	34.3 ± 1.5	6,892
Gestational age (weeks)	39.1 ± 1.6	751	39.1 ± 1.8	7,354
Male gender	50.7 %	385	51.2 %	3,775
Natural childbirth	53.1 %	399	50.2 %	3,733
Malformation at birth	1.5%	10	1.3%	87

[a] *High= Degree or higher*
