# Supplementary material for: Prenatal Influences on Size, Velocity and Tempo of Infant Growth: Findings from Three Contemporary Cohorts
Source: PLoS One. 2014 Feb 27;9(2):e90291. doi: 10.1371/journal.pone.0090291 (PMC3937389; doi:10.1371/journal.pone.0090291)
Supplement: Table S2 — “Minimally-adjusted” estimated coefficients and 95% confidence interval for the association between covariates and size and velocity parameters by cohorts. Gender- and gestational age-adjusted estimates of the association between covariates and size and velocity parameters obtained fitting the models to each whole cohort. (PDF) [file pone.0090291.s002.pdf]

TABLE S2. "Minimally-adjusted" estimated coefficients and 95% confidence interval for the association between covariates and size and velocity parameters by cohorts

	**GXXI** (n = 738)							
	Adjusted for gender				Adjusted for gender & gestational age			
	**Size**		**Velocity**		**Size**		**Velocity**	
	%	*95%CI*	%	*95%CI*	%	*95%CI*	%	*95%CI*
**Background**								
Maternal height	0.4	0.2; 0.6	0.02	-0.4; 0.4	0.4	0.2; 0.5	0.1	-0.3; 0.5
Maternal age	-0.01	-0.2; 0.2	0.3	-0.1; 0.8	0.1	-0.1; 0.2	0.2	-0.2; 0.6
Maternal parity [a]								
Nulliparous	0	--	0	--	0	--	0	--
Parous	3.5	1.2; 5.7	-2.9	-7.9; 1.9	3.2	1.2; 5.1	-2.4	-6.9; 2.0
Maternal education [b]								
Low	0-	--	0	--	0	--	0	--
Medium	2.7	0.0; 5.5	-1.9	-7.9; 4.1	3.4	1.0; 5.7	-2.7	-8.1; 2.7
High	0.2	-2.4; 2.8	-0.8	-6.6; 4.9	1.8	-0.5; 4.1	-3.8	-8.9; 1.4
**Intermediate**								
Pre-pregnancy BMI								
<18.5	-7.6	-13.2; -2.1	8.8	-3.5; 21.1	-4.4	-9.2; 0.5	2.5	-8.6; 13.6
18.5-24.99	0	--	0	--	0	--	0	--
25+	4.3	1.8; 6.8	-0.3	-5.8; 5.2	3.4	1.2; 5.6	1.1	-3.8; 6.1
Maternal Smoking [c]								
No	0	--	0	--	0	--	0	--
≤1st trimester	-4.1	-8.3; 0.1	5.9	-3.3; 15.1	-3.1	-6.7; 0.5	4.1	-4.1; 12.3
>1st trimester	-3.3	-6.5; -0.04	11.6	4.4; 18.7	-4.1	-6.8; -1.3	12.8	6.4; 19.1
Gestational diabetes								
No	0	--	0	--	0	--	0	--
Yes	1.4	-2.9; 5.8	-0.8	-10.5; 8.9	0.9	-3.0; 4.7	0.5	-8.2; 9.3
Gestational hypertension								
No	0	--	0	--	0	--	0	--
Yes	-8.3	-13.7; -2.9	19.4	7.6; 31.3	-3.0	-7.8; 1.9	9.7	-1.2; 20.6

	**NINFEA** (n = 2,925)							
	Adjusted by gender				Adjusted for gender & gestational age			
	**Size**		**Velocity**		**Size**		**Velocity**	
	%	*95%CI*	%	*95%CI*	%	*95%CI*	%	*95%CI*
**Background**								
Maternal height	0.4	0.4; 0.5	0.1	-0.2; 0.1	0.4	0.3; 0.5	0.02	-0.2; 0.2
Maternal age	-0.1	-0.2; 0.1	0.1	-0.2; 0.3	0.1	-0.02; 0.2	-0.2	-0.5; 0.04
Maternal parity [a]								
Nulliparous	0	--	0	--	0	--	0	--
Parous	1.2	0.02; 2.3	-1.6	-4.4; 1.1	3.0	2.0; 4.0	-5.7	-8.2; -3.2
Maternal education [b]								
Low	0.9	-1.5; 3.3	-1.6	-7.1; 4.0	-1.3	-3.5; 0.9	2.5	-3.0; 7.9
Medium	0	--	0	--	0	--	0	--
High	-0.2	-1.2; 0.9	-1.4	-3.9; 1.0	-0.4	-1.4; 0.6	-1.1	-3.5; 1.3
**Intermediate**								
Pre-pregnancy BMI								
<18.5	-4.6	-6.4; -2.8	5.3	1.0; 9.6	-5.2	-6.9; -3.6	6.8	2.6; 10.9
18.5-24.99	0	--	0	--	0	--	0	--
25+	2.5	1.2; 3.8	-3.0	-6.0; 0.01	2.1	0.9; 3.2	-1.9	-4.9; 0.9
Maternal Smoking [c]								
No	0	--	0	--	0	--	0	--
≤1st trimester	0.6	-3.2; 4.4	4.1	-4.8; 13.0	0.8	-2.7; 4.3	3.4	-5.2; 12.0
>1st trimester	-2.1	-4.1; -0.1	6.1	1.3; 10.9	-2.9	-4.8; -1.1	8.2	3.6; 12.9
Gestational diabetes								
No	0	--	0	--	0	--	0	--
Yes	0.4	-1.5; 2.3	-1.9	-6.2; 2.5	1.3	-0.4; 3.1	-3.7	-8.0; 0.5
Gestational hypertension								
No	0	--	0	--	0	--	0	--
Yes	-5.4	-7.3; -3.6	12.4	8.1; 16.7	-3.1	-4.7; -1.5	8.2	4.3; 12.1

	**GOCS** (n = 959)							
	Adjusted for gender				Adjusted for gender & gestational age			
	**Size**		**Velocity**		**Size**		**Velocity**	
	%	*95%CI*	%	*95%CI*	%	*95%CI*	%	*95%CI*
**Background**								
Maternal height	0.3	0.2; 0.4	-0.1	-0.4; 0.2	0.3	0.2; 0.4	-0.1	-0.4; 0.2
Maternal age	0.1	0.00; 0.2	-0.2	-0.5; 0.04	0.2	0.1; 0.3	-0.3	-0.6; - 0.1
Maternal parity [a]								
Nulliparous	0	--	0	--	0	--	0	--
Parous	1.6	0.2; 3.1	-3.6	-7.1; -0.1	2.3	0.9; 3.7	-4.9	-8.3; -1.6
Maternal education [b]								
Low	0.4	-1.2; 2.1	-4.2	-8.1; -0.2	0.3	-1.3; 1.9	-3.7	-7.4; 0.08
Medium	0	--	0	--	0	--	0	--
High	-0.7	-2.7; 1.2	-0.9	-5.6; 3.8	-0.9	-2.7; 1.0	-0.6	-5.0; 3.9
**Intermediate**								
Pre-pregnancy BMI								
<18.5	-4.1	-7.9; -0.3	-4.8	-13.9; 4.4	-3.6	-7.2; 1.0	-5.9	-14.7; 2.8
18.5-24.99	0	--	0	--	0	--	0	--
25+	1.6	-0.1; 3.4	-0.7	-5.0; 3.5	1.8	0.1; 3.5	-1.4	-5.4; 2.6
Maternal Smoking [c]								
No	0	--	0	--	0	--	0	--
≤1st trimester	-1.7	-4.2; 0.9	-0.5	-6.5; 5.6	-0.9	-3.3; 1.5	-3.1	-8.9; 2.7
>1st trimester								
Gestational diabetes								
No	0	--	0	--	0	--	0	--
Yes	2.4	-0.9; 5.7	-1.9	-9.9; 5.9	3.1	-0.1; 6.2	-3.7	-11.3; 3.4
Gestational hypertension								
No	0	--	0	--	0	--	0	--
Yes	0.7	-1.8; 3.2	0.6	-5.3; 6.5	1.5	-0.8; 3.9	-1.2	-6.9; 4.4

[a] *In GOCS child order was used as a proxy for parity*

[b] *GXXI: Low=≤9 years, Medium=≤12 years, High=Degree or higher; NINFEA: Low= ≤Secondary school, Medium=High school, High=Degree or higher ; GOCS: Low= None/Primary/Secondary school, Medium=High school, High=High School + technical education or higher*

[c] *In GOCS smoking during pregnancy was categorized as No/Rarely vs. Frequently*
